# A Retrospective Review of Men Referred to a Dedicated Erectile Dysfunction Clinic in Secondary Care

**DOI:** 10.7759/cureus.62537

**Published:** 2024-06-17

**Authors:** Muhammad Iqbal, Wail Mohamed, Mostafa Shendy, Anthony Shanahan, Martin Steggall, Gareth Brown

**Affiliations:** 1 Urology, Cwm Taf University Health Board, Royal Glamorgan Hospital Llantrisant, Cardiff, GBR; 2 Urology, Royal Glamorgan Hospital, Cardiff, GBR; 3 Urology, University of South Wales, Cardiff, GBR; 4 Urology, Cwm Taf University Health Board, Royal Glamorgan Hospital, Cardiff, GBR

**Keywords:** general practitioner, systemic review, primary care, secondary care, phosphodiesterase inhibitors, impotence, erectile dysfunction

## Abstract

Background and objectives

Erectile dysfunction (ED) is a multifactorial disease associated with many medical co-morbidities and risk factors commonly encountered in primary care. Initial management includes lifestyle changes and the treatment of any identifiable conditions. Guidelines exist recommending the assessment and management of sufferers with clear indications for referral to secondary care. With the outbreak of COVID-19, non-urgent medical services, including ED, were suspended, creating a significant waiting list for these patients. The aim of this study was to review the management of men in both primary and secondary care who had been referred to a dedicated ED service.

Materials and methods

A retrospective review of men referred to secondary care between June 2018 and April 2021 with ED was undertaken, reviewing whether the guidelines published by the National Institute for Health and Care Excellence (NICE) and GP Notebook for the assessment, initial treatment, and referral were followed by the primary care clinician. A secondary aim was to record the outcome of those men after review in a secondary care dedicated ED clinic.

Results

One hundred and forty-eight men were reviewed in the ED clinic, with 55 men (37.2%) requiring an intervention that was appropriate to have been delivered in primary care. The majority of those (76.3%) were successfully managed with a phosphodiesterase inhibitor. Of those treated in secondary care, almost 60% required a second-line therapy, such as a vacuum device or the administration of alprostadil, with 14 men (15%) necessitating the surgical implantation of a penile prosthesis.

Conclusion

With a rise in both the prevalence and incidence of ED, primary care physicians have a pivotal role in the screening and initial assessment of patients with ED, with evidence suggesting that a significant proportion can be successfully managed in this setting.

## Introduction

Erectile dysfunction (ED) is a common disorder of male sexual dysfunction that increases with age and in the presence of co-morbidities, such as hypertension and diabetes mellitus (DM). Defined as an inability to attain and/or maintain a penile erection sufficient for sexual activities, ED has a self-reported prevalence, as recorded in the Massachusetts Male Aging Study (MMAS) of 52% of men between the ages of 40 and 70 years. Of those men, 17.2% described mild ED, 25.2% moderate ED, and 9.6% severe ED [1,2]. The overall estimated incidence rate is 25.9 cases per 1000 man-years and rises with increasing age, ranging from 12.4 cases per 1000 man-years aged 40-49 years to 46.4 cases per 1000 man in men aged 60-69 years [3]. In the Cologne study of men aged 30-80 years, the prevalence of ED was 19.2%, with a steep age-related increase from 2.3% to 53.4% [4]. In a cross-sectional real-life study among men seeking first medical help for new-onset ED, one in four patients were younger than 40 years, with almost 50% of these men complaining of severe ED [5]. Not unexpectedly, ED has a significant impact on the quality of life of the patient and their partner, which negatively impacts self-confidence and self-esteem [6]. Causes of ED include organic that could be vascular (cardiac disease and previous pelvic surgery), neurogenic (multiple sclerosis, Parkinson’s disease, and spinal cord injury), anatomical (hypospadias, epispadias, micropenis, phimosis, and penile cancer), hormonal (DM, hypogonadism, and hyperprolactinemia) or drug-induced (alcohol, recreational drugs, cocaine, and steroids), and psychogenic or mixed. A common organic cause is a disorder of endothelial and smooth muscle cells of the penis. This endothelial dysfunction results in impaired blood flow and promotes atherosclerosis, which shares the same risk factors with cardiovascular, cerebrovascular, and peripheral vascular disease. Therefore, the importance of properly assessing ED is recognized with its potential early warning of an impending cardiovascular disease event [7].

Despite its high prevalence and significant impact on the mental well-being, quality of life, and health of its sufferer until relatively recently, ED was an underappreciated and under-treated condition until the availability of effective oral therapies. In the United Kingdom (UK), the initial assessment and management of ED is mostly performed in primary care with guidelines published online by the National Institute for Health and Care Excellence (NICE) and GP Notebook specifically for primary care clinicians [8,9].

Clinicians should undertake a full assessment to differentiate between an underlying organic or psychogenic cause while also taking into consideration signs and symptoms suggestive of associated conditions, such as diabetes, hypertension, hypogonadism, and hyperlipidemia. Risk factors for cardiovascular disease should be assessed, and blood pressure, heart rate, body mass index (BMI), waist circumference, focused genital examination, and when appropriate, a prostate examination should be performed. Investigations recommended should at least include lipid profile, fasting blood sugar/HbA1c, early morning 09.00-11.00 am testosterone levels, and PSA level, if appropriate. Additional investigation may be required, for example, thyroid function, hormone profile, and renal function, based on initial blood results and clinical findings. The use of validated scoring systems, such as the International Index of Erectile Function (IIEF) questionnaire, Erection Hardness Score (EHS), and Sexual Health Inventory for Men (SHIM) questionnaire, can be a useful adjunct in assessing the severity of ED and the efficacy of treatments. The SHIM scores range from 1 to 21, with a score of 1-7 corresponding with severe ED, 8-11 moderate ED, 12-16 mild to moderate ED, and 17-21 indicating mild ED [10].

Treatments should be commenced in primary care and include lifestyle modifications (such as weight loss, participating in regular exercise, smoking cessation, and reducing alcohol intake), correcting reversible conditions (e.g., hypertension, diabetes, and dyslipidemia), and when possible, changing medication known to precipitate ED. Primary care, therefore, has a key role in ensuring that underlying issues are addressed, risk factors identified and treated, lifestyle changes implemented, and psychological conditions managed.

Both NICE and GP Notebook currently recommend that unless contraindicated phosphodiesterase inhibitors (PDE5i’s) be prescribed in primary care with patients trialing eight doses with sexual stimulation at maximum dose before deeming them non-responders. For optimal results, patients must be counseled on how and when to take the medication, its possible interactions and potential side effects, and with full engagement of partners. Prescribing information for both clinicians and patients can be accessed through websites such as NICE and the British Association of Urological Surgeons (BAUS). A follow-up consultation is required to reinforce lifestyle changes, assess treatment effectiveness, and, if unsuccessful, ensure that the PDE5i was taken correctly or dose altered as required. Only when first-line therapies are ineffective or contraindicated or specialized investigations are required should the patient be referred to secondary care, for example, urology for consideration of second-line ED treatments or when penile abnormalities co-exist, endocrine for hypogonadism or uncontrolled diabetes, and psycho-sexual counseling if underlying refractory psychogenic ED.

## Materials and methods

Ethical consideration

All patient information was retrospectively collected, anonymized, and stored in a secured database within the health board’s IT systems.

Study aims

The primary aim of this study was to review whether patients received appropriate ED assessment and management in primary care following the guidance published by the NICE and GP Notebook before being referred to an ED service in secondary care. An additional aim was to determine the treatment outcomes of those patients seen in secondary care and consider whether all referrals were appropriate and in accordance with the recommendations of the NICE and GP Notebook.

Study design

This was a retrospective review of all National Health Service (NHS) patients referred by their GP between 2018 and 2021 to an ED service run by a urology department in a single institute in the UK. GP referrals to the ED clinic are received and vetted by the on-call urology consultant daily. If it is documented in the referral letter that the patients have been assessed, including blood tests, and managed following the GP guidelines, they are then reviewed in a dedicated consultant “run” ED service [8,9]. Any patients with ED who had not been fully assessed or investigated or trialed on at least two PDE5i’s at maximum dose were referred back to their GP with an accompanying letter of explanation and recommendation and were excluded from the study. Patients were also excluded if they had other known co-existing conditions, such as Peyronie’s disease, phimosis, and gender reassignment, or were suspected of other urological conditions, such as prostate or penile cancer or hematuria and were instead seen in the appropriate setting.

In the ED clinic, patients were asked to complete a proforma that included an IIEF questionnaire, an EHS self-assessment, and a bother score (Tables 1-3), with blood pressure, heart rate, BMI, and waist circumference recorded before they were reviewed by a clinician. In addition to a history and examination, the previous use of any ED treatments is noted, and if the medication was taken correctly. Baseline early morning fasted blood tests, including HbA1c, lipid profile, testosterone, and, if appropriate, a PSA, were requested for patients if not performed in the preceding 12 months. Patients were commenced on treatments and reviewed to assess their effectiveness and the results of any blood work. All men were followed up until successfully treated or declined further intervention.

**Table 1:** IIEF questionnaire The IIEF-5 score is the sum of questions 1-5. The lowest score is 5, and the highest score is 25. If the score is 21 or less, you may be showing signs of ED. Please speak with the person who gave you this form or ask your GP for advice. IIEF, International Index of Erectile Function; ED, erectile dysfunction

Over the past six months	Score				
	1	2	3	4	5
How do you rate your confidence that you could get and keep an erection?	Very low	Low	Moderate	High	Very high
When you had erections with sexual stimulation, how often were your erections hard enough for penetration?	Almost never or never	Much less than half the time	About half the time	Much more than half the time	Almost always or always
During sexual intercourse, how often were you able to maintain your erection after you had penetrated (entered) your partner?	Almost never or never	Much less than half the time	About half the time	Much more than half the time	Almost always or always
During sexual intercourse, how difficult was it to maintain your erection to the completion of intercourse?	Extremely difficult	Very difficult	Difficult	Slightly difficult	Not difficult
When you attempted sexual intercourse, how often was it satisfactory for you?	Almost never or never	Much less than half the time	About half the time	Much more than half the time	Almost always or always

**Table 2:** Subjective bother score

	Delighted	Pleased	Mostly satisfied	Mixed (about equally satisfied)	Mostly dissatisfied	Unhappy	Terrible
If you were to spend the rest of your life with your erection trouble the way it is now, how would you feel about that?	0	1	2	3	4	5	6

**Table 3:** EHS self-assessment EHS, erection hardness score

How would you rate the hardness of your erection?	
0	Penis does not enlarge
1	Penis is larger but not hard
2	Penis is hard but not hard enough for penetration
3	Penis is hard enough for penetration but not completely hard
4	Penis is completely hard and fully rigid

## Results

Between June 2018 and April 2021, a total of 148 patients referred by their GP with ED fulfilled the inclusion criteria and were included in the review. The age range of men referred was between 29 and 84 years old, with the commonest age range of 60-70 years old. The number of men with associated pre-existing conditions is shown in Table 4.

**Table 4:** The prevalence of pre-existing medical conditions or ED risk factors in the men referred ED, erectile dysfunction

Comorbidities	Percentage of men (%)
Diabetes mellitus	36
Hypertensive	20
Ischemic heart disease	18
Obesity	6.8
Hyperlipidemia	6.8
Pelvic surgery/radiotherapy	8.1
Hypogonadal	6.8
Current smoker	18
Ex-smoker	24

Only 15 patients (10.1%) had been advised by their GPs about weight reduction, regular exercise, smoking cessation, and reducing alcohol intake. Upon questioning, 133 men (89.9%), however, had not been counseled by their GP about the benefits of lifestyle modifications.

Despite the guidelines specifying that blood tests be performed to assess for an underlying cause or cardiovascular risk factors, only 57 men (38.5%) had the recommended blood tests performed and documented by their GP. Eighty-one men had testosterone, HbA1c, lipid profile, and PSA tests through the ED clinic, with 10 patients having had blood tests incidentally performed by other specialties or declined further treatment. Of those men prescribed medication to treat known hypogonadism, dyslipidemia, or diabetes, 33 (22.3%) were found to still have deranged blood work, with 22 identified through the ED service and 11 through their GP.

Of the 148 patients who attended the ED clinic, 36 may have been managed in primary care, requiring only an alteration to their PDE5i treatment to treat their ED successfully. Of those, seven men required an increase in dose, 17 were switched to a daily dosing regimen, eight men were prescribed an alternate PDE5i, and four needed education on correct usage (either on an empty stomach or in conjunction with sexual stimulation). Six men denied having trialed a PDE5i despite being documented in the referral letter. A further nine men seen in the ED service were requesting psychosexual counseling, one man needed advice on diabetic lifestyle changes, two declined any treatment, and one denied any symptoms of ED. Therefore, 55 (37.2%) could have been managed in primary care (Table 5).

**Table 5:** The percentage of men reviewed in an ED clinic with treatment outcomes that may have been delivered in primary care ED, erectile dysfunction; PDE5i, phosphodiesterase inhibitor

Treatment outcomes in primary care	Percentage of men (%)
Alteration in PDE5i	
Increase dose	4.7
Changed to daily prescription	11.4
Switch to another PDE5i	5.4
Education in medication usage	2.7
Newly commenced on PDE5i	4.05
Psychosexual counseling	6.08
Additional conservative advice	0.6
Declined treatment	1.35
No ED	0.6

Ninety-three men (62.8%) were refractory or contraindicated to PDE5i treatment, with the majority requiring either second-line or third-line therapies delivered in secondary care. Of these, 55 men (59.1%) were treated with a second-line therapy, with 28 men (30.1%) successfully using a vacuum erection aid and 27 men (29%) relying on the drug alprostadil. Only 14 men (15.1%) progressed to the insertion of a penile prosthesis. The remaining men were found to have undiagnosed conditions not noted by their referring GP, such as Peyronie’s disease, phimosis, or retrograde ejaculation (Table 6).

**Table 6:** The number of men who required secondary care interventions through a dedicated ED clinic with their treatment outcomes ED, erectile dysfunction

Treatment outcomes in secondary care	Number of men
Vacuum erection device	28
Alprostadil (urethral pellet/intracavernosal injection)	27
Testosterone replacement therapy	8
Penile prosthesis	14
Psychogenic ED (NPT)	5
Peyronie’s disease	5
Retrograde ejaculation	1
Phimosis	2
Declined 2nd or 3rd line therapies	3

## Discussion

Our results suggest that more than a third of patients, or 55 men (37.1%), who were referred to secondary care required only changes in their management that could have been delivered in primary care within the scope of the current guidelines. Of those, 42 men (76.3%) were treated successfully with a PDE5i, necessitating only their initiation or modification. It was beyond the scope of this study to examine what the reasons were as to why these patients were not fully managed in primary care, but patient factors, consultation time pressures, inaccuracies in the referral letter, and perhaps inconsistencies in vetting referrals within the urology department are likely contributing factors. In total, 91 consultations could have been avoided in secondary care, which would have had cost-saving implications or allowed the review of more appropriate patients in the dedicated ED clinic. 

The finding of deranged blood tests in our review pertinent to pre-existing medical conditions relevant to ED in more than 20% of patients suggests that there may be the capacity to further improve treatments in primary care, which may have a bearing not only on the immediate concern of ED but also on long term health. Steggall et al. suggested that clinical management of ED can be difficult to achieve with patients having uncorrected underlying disorders. Primary care colleagues should ideally have the time and resources to identify and manage men with ED. This would be the most cost-effective way forward and avoid unnecessary follow-ups or referrals to secondary care [11].

In primary care, current guidelines advocate that those men are not only treated for their ED but fully evaluated and assessed for an underlying cause and the presence of co-existing risk factors. These well-established guidelines provide a standard of care recommending that all men presenting with ED should undergo a CVD risk assessment, testosterone level for assessment of hypogonadism, lipid profile, HbA1c, and PSA levels if appropriate. An approach to managing and treating ED in primary care is advised with referral to secondary care proposed for young patients with primary ED, those requiring specialist investigations, or those who are either refractory or contraindicated to the use of phosphodiesterase inhibitors [12]. Delays in treatment can have profound effects on the mental health of individuals or miss the prospect of offering conservative measures for ED caused by certain lifestyle factors such as smoking, alcohol, obesity, sedentary, or poor diet. Early intervention may potentially reverse this temporary form of ED as once the underlying disease is established, dependence on medical management will be long-standing [13]. A delay in treating ED can also have a significant negative impact on the man’s confidence, with the fear of failure developing into the potential for avoidance of intimacy with a resultant breakdown in the relationship. In a paper by Sadovsky, it was noted that primary care clinicians are more likely to build rapport and develop personal relationship with their patients, which may make discussing and resolving sexual problems easier to navigate [14]. Rosenberg suggested that the evaluation of ED can be delivered in primary care with treatment in most circumstances initiated regardless of the ED etiology and during investigation [15].

Between 2019 and 2021, the impact of COVID-19 on the provision of health services was profound. A restructuring of health care resources was required to ensure safe clinical care without compromising the safety and health of both staff and patients. This meant that numerous services deemed non-essential were temporarily suspended. This included the provision of an ED service, with many patients having to wait a long time either to be seen in primary care or reviewed in secondary care. The legacy of COVID-19 created a significant backlog of both new and follow-up consultations that proved challenging for the health service. These pressures were experienced across both primary and secondary care. In our institution, we were faced with an immediate waiting time of 24 months for new patients once the ED service was “re-opened”. Other specialties also encountered similar waiting times for their services, which had been deferred during the COVID-19 pandemic. These demands triggered a review of the referral of men with ED from primary care as well as the outcome of their management in secondary care to try and identify any measures that could be put in place to help meet the now high demands on the service.

The main limitation of this review is the potential for several types of bias. This is a retrospective review of the GP referrals vetted by five different urology consultants with the potential for selection bias. Differences in patient education and socioeconomic background may lead to variations in expectations and engagement with lifestyle and therapeutic measures. In addition, some men may have difficulty engaging with primary care if reviewed by female clinicians. Finally, withholding symptoms due to embarrassment may lead to erroneous assumptions or inaccurate referral information.

ED is a common disorder with a rising incidence. It is a multifactorial disease that can be a symptom of underlying cardiovascular disease, diabetes, testosterone deficiency, dyslipidemia, and/or hypertension. For the majority of men with ED, their first approach to a health provider is in primary care, either as the main complaint or disclosed during the consultation. Men historically have always been poor in seeking medical advice, so this first point of contact provides a unique opportunity to assess or “screen” for new underlying conditions. Primary care physicians provide comprehensive care, and as many of the medical problems associated with ED are commonly encountered and managed in primary care, they are best suited to recognizing and treating potentially reversible conditions or reducing the progression of ill health.

## Conclusions

As ED is associated with a wide variety of etiologies and a diverse range of treatments, a collaborative approach to managing ED between primary and secondary care may be required to avoid unnecessary consultations and ensure that the appropriate services are delivered in the correct setting. With a rise in both the prevalence and incidence of ED, primary care physicians have a pivotal role in the screening and initial assessment of patients with ED, with evidence suggesting that a significant proportion can be successfully managed within general practice.
